# Supplementary material for: COVID-19 Vaccine Coverage and Sociodemographic, Behavioural and Housing Factors Associated with Vaccination among People Experiencing Homelessness in Toronto, Canada: A Cross-Sectional Study
Source: Vaccines (Basel). 2022 Aug 3;10(8):1245. doi: 10.3390/vaccines10081245 (PMC9412474; doi:10.3390/vaccines10081245)
Supplement: Supplementary file 1 [file vaccines-10-01245-s001.zip › vaccines-1809922-supplementary.pdf]

Supplementary Materials

Table S1 – STROBE Checklist of items relevant for *cross-sectional studies*

	Item No	Recommendation	Page No
**Title and abstract**	1	(*a*) Indicate the study's design with a commonly used term in the title or the abstract	1
		(*b*) Provide in the abstract an informative and balanced summary of what was done and what was found	1
**Introduction**			
Background/rationale	2	Explain the scientific background and rationale for the investigation being reported	1
Objectives	3	State specific objectives, including any prespecified hypotheses	2
**Methods**			
Study design	4	Present key elements of study design early in the paper	2
Setting	5	Describe the setting, locations, and relevant dates, including periods of recruitment, exposure, follow-up, and data collection	2
Participants	6	(*a*) Give the eligibility criteria, and the sources and methods of selection of participants	3
Variables	7	Clearly define all outcomes, exposures, predictors, potential confounders, and effect modifiers. Give diagnostic criteria, if applicable	3
Data sources/ measurement	8*	For each variable of interest, give sources of data and details of methods of assessment (measurement). Describe comparability of assessment methods if there is more than one group	2, 3
Bias	9	Describe any efforts to address potential sources of bias	N/A
Study size	10	Explain how the study size was arrived at	3
Quantitative variables	11	Explain how quantitative variables were handled in the analyses. If applicable, describe which groupings were chosen and why	3, 4
Statistical methods	12	(*a*) Describe all statistical methods, including those used to control for confounding	3, 4
		(*b*) Describe any methods used to examine subgroups and interactions	N/A
		(*c*) Explain how missing data were addressed	4
		(*d*) If applicable, describe analytical methods taking account of sampling strategy	3, 4
		(*e*) Describe any sensitivity analyses	N/A
**Results**			
Participants	13*	(a) Report numbers of individuals at each stage of study—eg numbers potentially eligible, examined for eligibility, confirmed eligible, included in the study, completing follow-up, and analysed	4

		(b) Give reasons for non-participation at each stage	4
		(c) Consider use of a flow diagram	4
Descriptive data	14*	(a) Give characteristics of study participants (eg demographic, clinical, social) and information on exposures and potential confounders	4, 5, 6
		(b) Indicate number of participants with missing data for each variable of interest	5, 6
Outcome data	15*	Report numbers of outcome events or summary measures	5, 6
Main results	16	(*a*) Give unadjusted estimates and, if applicable, confounder-adjusted estimates and their precision (eg, 95% confidence interval). Make clear which confounders were adjusted for and why they were included	7, 4
		(*b*) Report category boundaries when continuous variables were categorized	N/A
		(*c*) If relevant, consider translating estimates of relative risk into absolute risk for a meaningful time period	N/A
Other analyses	17	Report other analyses done—eg analyses of subgroups and interactions, and sensitivity analyses	N/A
**Discussion**			
Key results	18	Summarise key results with reference to study objectives	8
Limitations	19	Discuss limitations of the study, taking into account sources of potential bias or imprecision. Discuss both direction and magnitude of any potential bias	9
Interpretation	20	Give a cautious overall interpretation of results considering objectives, limitations, multiplicity of analyses, results from similar studies, and other relevant evidence	8, 9
Generalisability	21	Discuss the generalisability (external validity) of the study results	8, 9
**Other information**			
Funding	22	Give the source of funding and the role of the funders for the present study and, if applicable, for the original study on which the present article is based	10

*Give information separately for exposed and unexposed groups.

Note: An Explanation and Elaboration article discusses each checklist item and gives methodological background and published examples of transparent reporting. The STROBE checklist is best used in conjunction with this article (freely available on the Web sites of PLoS Medicine at http://www.plosmedicine.org/, Annals of Internal Medicine at http://www.annals.org/, and Epidemiology at http://www.epidem.com/). Information on the STROBE Initiative is available at www.strobe-statement.org.

Table S2 – Full variable definitions

The table below includes all variables included in this analysis. Included covariates are either shown in existing literature to have associations with vaccine-seeking behaviour or are known to affect risk for COVID-19 related adverse health outcomes (which may also, indirectly, affect vaccine-seeking behaviour). In all instances, measures were collected based on a Settler Canadian (Western) understanding of health-related factors and associated prevention guidelines appropriate during the COVID-19 pandemic.

Variable	Definition	Source
**Outcome**		
**COVID-19 vaccination**	Participant's receipt of any Health-Canada approved COVID-19 vaccine by the interview date. Answers include Yes (1+ dose), or No.<br>Category is determined using participant self-report and participant biological sample results.	Self-report: Statistics Canada's COVID-19 Vaccination Coverage Survey[1] ("Have you been vaccinated against COVID-19?" and "How many doses of the COVID-19 vaccine have you received so far?")<br><br>Biological data: ELISA test on blood sample.
**Covariates**		
Age	Participant's age as of the date of the survey. Calculated using the date of the survey and the participant's self-reported date of birth.	At Home/Chez Soi Study[2]
Gender	Participant's self-reported gender, as of the interview date. Answers categorized into 'male', 'female', 'other' and 'refused/don't know'. 'Refused/don't know' category is imputed in multivariable analysis, and not included in bivariate comparisons.	Modifed from At Home/Chez Soi Study[2] ("What is your gender? Do you identify as: [Male] [Female] [Non-Binary, Gender Queer, Agender, or a Similar Identity] [Two-Spirit] [Other]")
Indigenous	Whether or not the participant self-identifies as Indigenous as of the interview date. Indigenous status includes First Nations, Métis, Inuit or similar groups. Answers include 'Yes', 'No', 'Refused/Don't know'. 'Refused/don't know' category is imputed in multivariable analysis, and not included in bivariate comparisons	CIHI Standards for Race-Based and Indigenous Identity Data Collection and Health Reporting in Canada[3] ("Do you identify as First Nations, Metis and/or Inuk/Inuit?")
Race	Participant's self-reported race category as of the interview date. Answers include 'White', 'Black', 'Indigenous', 'Other/multiracial' (which includes 'Arab/Middle Eastern/West Asian', 'Latin American', 'East/Southeast Asian', 'South Asian/Indo-Carribean' and 'More than one race category or mixed race'), and 'Refused/don't know'. 'Refused/don't know' category is imputed in multivariable analysis, and not included in bivariate comparisons.	CIHI Standards for Race-Based and Indigenous Identity Data Collection and Health Reporting in Canada[3] (Which race categories best describe you?")

Citizenship	Participant's self-reported citizenship status, as of the interview date. Answers include 'Citizen', 'Landed immigrant' (also known as 'Permanent resident'), 'Refugee', 'Temporary/Other' and 'Refused/Don't know'). 'Refused/don't know' category is imputed in multivariable analysis, and not included in bivariate comparisons.	At Home/Chez Soi Study[2] ("What is your current status in Canada?")
Education level	Participant's self-reported highest level of completed education, as of the interview date. Answers include 'less than high school' (secondary school), 'high school', 'any-post-secondary' (which combines 'vocational/technical school', 'college/university', 'graduate/professional school'), or 'Refused/Don't know'. 'Refused/don't know' category is imputed in multivariable analysis, and not included in bivariate comparisons.	At Home/Chez Soi Study[2] ("What is the highest level of education you have completed?")
Paid work since March 2020	Participant's self-reported work experience since the start of the pandemic (March 2020). Work includes any form of paid activity. Answers categorized into 'Yes' and 'No/Refused/Don't know'.	New question ("Have you done any paid work since March 1 2020?")
Primary housing type in past 90 days	Housing type representing the participant's most common (greatest proportion of time) living situation in the past 90 days immediately preceding the interview date. Housing types include 'Homeless shelter', 'Physical distancing hotel' (hotels temporarily commissioned as shelters for physical distancing during the pandemic), and Other (including varied settings such as 'Encampment', 'Street', 'Jail', 'Staying with friends and family', or 'Own home'). This variable is computed using the self-reported housing history instrument in the survey, which asks Participants to recall their housing episodes from most recent to least recent, going at least 90 days back.	At Home/Chez Soi Study[2]
Presence of chronic conditions	Whether or not the participant has at least one chronic condition that was diagnosed by a physician (self-reported by the participant as of the interview date). The chronic conditions include: Hypertension, diabetes, asthma, chronic lung disease, chronic heart disease, stroke, chronic kidney disease, chronic neurological disorder, liver disease, cancer, HIV/AIDS or an immunological disease other than HIV/AIDS	At Home/Chez Soi Study[2] ("Have you ever been diagnosed by a physician with any of the following chronic medical conditions?")
Body mass index category	The participant's body mass index category, calculated using the participant's self-reported height and weight. Categories include 'underweight/normal' (representing BMI values <25), 'overweight' (representing BMI values between 25 and 30), 'obese' (representing BMI values over 30), and 'missing' (representing those not answering the height and/or weight question or whose answer is not biologically possible). 'Missing' category is not included in bivariate comparisons	At Home/Chez Soi Study[2] ("What is your current height? An estimate is fine" and "What is your current weight? An estimate is fine")

Influenza vaccine in past season	The participant's self-reported receipt of an influenza vaccine in the past season (in fall 2020/winter 2021). Answers include Yes or No/Refused/Don't know.	New question ("Have you received the flu shot this fall or winter (October 2021 or later?")
Observance of public health guidelines: wearing a face mask in public places	The participant's self-reported adherence to public health guidelines active in Ontario at the time of the interview (specifically: the degree to which the participant wears a face mask when in public places). Answers include 'Low' (including Never/rarely/occasionally), 'High' (including 'Often/Always') and 'Refused/Don't know. 'Refused/don't know' category is imputed in multivariable analysis, and not included in bivariate comparisons.	CDC Data Foundation COVID Impact Survey[4] ("How often have you… worn a face mask in public places"?)
**Observance of public health guidelines: distancing in public places**	The participant's self-reported adherence to public health guidelines active in Ontario at the time of the interview (specifically: the degree to which the participant practices physical distancing while in public places). Answers include 'Low' (including Never/rarely/occasionally), 'High' (including 'Often/Always') and 'Refused/Don't know. 'Refused/don't know' category is imputed in multivariable analysis, and not included in bivariate comparisons.	CDC Data Foundation COVID Impact Survey[4] ("How often have you… practiced physical distancing in public places"?)
**Observance of public health guidelines: avoiding crowded places or gatherings**	The participant's self-reported adherence to public health guidelines active in Ontario at the time of the interview (specifically: the degree to which the participant avoids crowded places or gatherings). Answers include 'Low' (including Never/rarely/occasionally), 'High' (including 'Often/Always') and 'Refused/Don't know. 'Refused/don't know' category is imputed in multivariable analysis, and not included in bivariate comparisons.	CDC Data Foundation COVID Impact Survey[4] ("How often have you… avoided crowded places or gatherings"?)
**Observance of public health guidelines: washing hands several times per day**	The participant's self-reported adherence to public health guidelines active in Ontario at the time of the interview (specifically: the degree to which the participant washes their hands with soap/hand sanitizer several times per day). Answers include 'Low' (including Never/rarely/occasionally), 'High' (including 'Often/Always') and 'Refused/Don't know. 'Refused/don't know' category is imputed in multivariable analysis, and not included in bivariate comparisons.	CDC Data Foundation COVID Impact Survey[4] ("How often have you… washed hands with soap or hand sanitizer several times per day"?)

References:

1. Statistics Canada. COVID-19 Vaccination Coverage Survey (CVCS). Available online : https://www23.statcan.gc.ca/imdb/p2SV.pl?Function=getSurvey&SDDS=5347#a3 (Accessed July 22 2022).
2. Goering PN, Streiner DL, Adair C, et al. The At Home/Chez Soi trial protocol: a pragmatic, multi-site, randomized controlled trial of a Housing First intervention for homeless individuals with mental illness in five Canadian cities. *BMJ Open*. 2011; 1(2).
3. Canadian Institute for Health Information. Proposed Standards for Race-Based and Indigenous Identity Data Collection and Health Reporting in Canada. Ottawa, ON: CIHI; 2020. Available online: www.cihi.ca/sites/default/files/rot/proposed-standard-for-race-based-data-en.pdf (Accessed July 22, 2022).
4. Hutchins HJ, Wolff B, Leeb R, et al. COVID-19 Mitigation Behaviors by Age Group – United States, April-June 2020. *MMWR Morb Mortal Wkly Rep*. 2020; 69: 1584–1590. doi: http://dx.doi.org/10.15585/mmwr.mm6943e4external icon.

Table S3 – COVENANT vaccine type received by vaccinated participants, by dose number

Vaccine Type	First dose (n=585)	Second dose (n=463)
Pfizer	289 (49.4%)	229 (49.5%)
Moderna	161 (27.5%)	153 (33.0%)
Astra Zeneca/Covishield	37 (6.3%)	7 (1.5%)
Johnson & Johnson	3 (0.5%)	1 (0.2%)
Unknown	95 (16.2%)	73 (15.8%)

Table S4 – COVENANT participant characteristics at baseline, overall and by vaccination dose number

Participant characteristics		Total (n=728)	No dose (n=143)	1 dose (n=122)	2+ dose (n=463)
Age	mean (SD)	46.1 (14.7)	39.1 (13.8)	47.8 (14.4)	49.1 (14.4)
	median (IQR)	46 (34-58)	36 (28-48)	42 (32-5)	50 (38-60)
Self-reported gender	% Male	481 (66.1%)	83 (58.0%)	81 (66.4%)	317 (68.5%)
	% Female	228 (31.3%)	56 (39.2%)	36 (29.5%)	136 (29.4%)
	% Other	17 (2.3%)	4 (2.8%)	4 (3.3%)	9 (1.9%)
	Refused/Don't know[1]	2 (0.3%)	0 (0%)	1 (0.8%)	1 (0.2%)
Identifies as Indigenous	Yes	75 (10.30%)	12 (8.39%)	15 (12.3%)	48 (10.4%)
	No	631 (86.68%)	128 (89.51%)	102 (83.6%)	401 (86.6%)
	Refused/Don't know[1]	22 (3.02%)	3 (2.10%)	5 (4.1%)	14 (3.0%)
Self-reported race	Black	159 (21.84%)	47 (32.87%)	67 (54.9%)	233 (50.3%)
	Indigenous	27 (3.71%)	5 (3.50%)	25 (20.5%)	87 (18.8%)
	White	353 (48.49%)	53 (37.06%)	4 (3.3%)	18 (3.9%)
	Other/multiracial	156 (21.43%)	31 (21.68%)	20 (16.4%)	105 (22.7%)
	Refused/Don't know[1]	33 (4.53%)	7 (4.90%)	6 (4.9%)	20 (4.3%)
Citizenship status	Citizen	556 (76.4%)	112 (78.3%)	93 (76.2%)	351 (75.8%)
	Landed immigrant	90 (12.4%)	11 (7.7%)	11 (9.0%)	68 (14.7%)
	Refugee	55 (7.6%)	11 (7.7%)	10 (8.2%)	34 (7.3%)
	Temporary/Other	20 (2.8%)	6 (4.2%)	5 (4.1%)	9 (1.9%)
	Refused/Don't know[1]	7 (1.0%)	3 (2.1%)	3 (2.5%)	1 (0.2%)
Highest level of education completed	Less than high school	206 (28.3%)	33 (23.1%)	33 (27.1%)	140 (30.2%)
	High school	251 (34.5%)	65 (45.5%)	52 (42.6%)	134 (28.9%)
	Any post-secondary	262 (36.0%)	44 (30.8%)	34 (27.9%)	184 (29.7%)
	Refused/Don't know[1]	9 (1.2%)	1 (0.7%)	3 (2.5%)	5 (1.1%)
Engaged in paid work since March 2020		165 (22.7%)	44 (30.8%)	31 (25.4%)	90 (19.4%)
Primary housing type in past 90 days	Homeless shelter	287 (39.4%)	49 (34.3%)	44 (36.1%)	194 (41.9%)
	Physical distancing hotel	328 (45.1%)	60 (42.0%)	44 (36.1%)	224 (48.4%)
	Other setting	113 (15.5%)	34 (23.8%)	34 (27.9%)	45 (9.7%)
Presence of chronic condition(s)[2]		350 (48.1%)	57 (39.9%)	53 (43.4%)	240 (51.8%)
Body mass index category	Underweight/normal	333 (45.7%)	61 (42.7%)	62 (50.8%)	210 (45.4%)
	Overweight	226 (31.0%)	51 (35.7%)	27 (22.1%)	148 (32.0%)
	Obese	140 (19.2%)	27 (18.9%)	23 (18.9%)	90 (19.4%)
	Missing[1]	29 (4.0%)	4 (2.8%)	10 (8.2%)	15 (3.2%)
Influenza vaccine in past season		217 (29.8%)	13 (9.1%)	30 (23.6%)	174 (37.6%)
Observance of Public Health Guidelines: wear face mask in public	Low (never/rarely/occasionally)	93 (12.9%)	30 (21.1%)	18 (14.8%)	45 (9.7%)
	High (often/always)	630 (87.1%)	112 (78.9%)	102 (83.6%)	416 (89.9%)
	Refused/Don't know[1]	5 (0.7%)	1 (0.7%)	2 (1.6%)	2 (0.4%)
Observance of Public Health Guidelines: distancing in public places	Low (never/rarely/occasionally)	103 (14.3%)	26 (18.4%)	22 (18.0%)	55 (11.9%)
	High (often/always)	615 (85.7%)	115 (81.6%)	96 (78.7%)	404 (87.3%)
	Refused/Don't know[1]	10 (1.8%)	2 (1.4%)	4 (3.3%)	4 (0.9%)

Observance of Public Health Guidelines: avoid crowded places or gatherings	Low (never/rarely/occasionally)	136 (19.1%)	36 (25.7%)	28 (23.0%)	72 (15.6%)
	High (often/always)	577 (80.9%)	104 (74.3%)	92 (75.4%)	381 (82.3%)
	Refused/Don't know[1]	15 (2.1%)	3 (2.1%)	2 (1.6%)	10 (2.2%)
Observance of Public Health Guidelines: wash hands with soap or sanitizer several times per day	Low (never/rarely/occasionally)	72 (10.0%)	17 (12.0%)	13 (10.7%)	42 (9.1%)
	High (often/always)	651 (90.0%)	125 (88.0%)	108 (88.5%)	418 (90.3%)
	Refused/Don't know[1]	5 (0.7%)	1 (0.7%)	1 (0.8%)	3 (0.7%)
